# Complete Sequences of the Human T-Cell Leukemia Virus Type 1 Proviral Genomes from Newly Established Adult T-Cell Leukemia Cell Lines in Oita Prefecture, Japan

**DOI:** 10.1128/genomeA.00090-18

**Published:** 2018-06-21

**Authors:** Takuro Fukumoto, Emi Ikebe, Masao Ogata, Kazuhiro Kohno, Madoka Kuramitsu, Yusuke Sato, Nichole Fife, Takashi Matsumoto, Takaaki Yahiro, Masanori Ikeda, Shuichi Kusano, Akihiko Okayama, Mitsuo Hori, Naoki Hijiya, Yoshiyuki Tsukamoto, Yuka Hirashita, Masatsugu Moriyama, Kamruddin Ahmed, Hiroo Hasegawa, Akira Nishizono, Masumichi Saito, Hidekatsu Iha

**Affiliations:** a Department of Microbiology, Faculty of Medicine, Oita University, Yufu, Japan; b Department of Safety Research on Blood and Biological Products, National Institute of Infectious Diseases, Musashimurayama, Tokyo, Japan; c Department of Medical Oncology & Hematology, Faculty of Medicine, Oita University, Yufu, Japan; d Division of Persistent and Oncogenic Viruses, Center for Chronic Viral Diseases, Graduate School of Medical and Dental Sciences, Kagoshima University, Kagoshima, Japan; e Department of Rheumatology, Infectious Diseases and Laboratory Medicine, Faculty of Medicine, University of Miyazaki, Kiyotake, Miyazaki, Japan; f Department of Hematology, Ibaraki Prefectural Central Hospital, Kasama, Ibaraki, Japan; g Department of Molecular Pathology, Faculty of Medicine, Oita University, Yufu, Japan; h Department of Pathobiology and Medical Diagnostics, Faculty of Medicine and Health Sciences, University Malaysia Sabah, Kota Kinabalu, Malaysia; i Department of Laboratory Medicine, Nagasaki University Graduate School of Biomedical Sciences, Nagasaki, Japan

## Abstract

We report two complete proviral genome sequences of human T-cell leukemia virus type 1 (HTLV-1) isolated from the peripheral blood specimens of acute type adult T-cell leukemia (ATL) patients in Oita Prefecture, Japan.

## GENOME ANNOUNCEMENT

Human T-cell leukemia virus type 1 (HTLV-1) is a retrovirus belonging to the family Retroviridae, genus Deltaretrovirus, and an etiological agent for adult T-cell leukemia (ATL) (1) and neurological disorders termed HTLV-1-associated myelopathy/tropical spastic paraparesis (HAM/TSP) (2). Current studies indicate that there are more than 20 million HTLV-1 carriers worldwide, and 5% of these carriers will develop ATL (3). HTLV-1 produces two oncogenic proteins, Tax and HBZ. While Tax is essential for initial immortalization of HTLV-1-infected cells, HBZ is more important for the onset of ATL symptoms through its immune compromising and cellular proliferating function. Messenger RNAs of both oncoproteins are transcribed from the 3ʹ regions of HTLV-1 provirus genome where each open reading frame is located in either the sense or antisense part (4). The expression of Tax is frequently canceled by deletion (5) or epigenetic modulation (i.e., methylation) (6) of the proviral 5ʹ regions. It is widely recognized that Tax is less important for ATL onset, and a previous report indicated Tax’s disappearance in the freshly isolated HTLV-1-positive peripheral blood cells (PBLs) from 60% of ATL cases (6).

Here, we report the complete HTLV-1 provirus genome sequence of OATL9, a novel HTLV-1/EBV-infected B-cell line isolated from the acute type ATL patient (7). The study was approved through the institutional review board of the Oita University Faculty of Medicine, approval number 267. Two provirus clones within OATL9 were located in chromosomes 1 and 15, respectively, and amplified by a PCR method that has been described (8). The amplicons were purified using the PCR cleanup gel extraction kit (Macherey-Nagel) and sequenced directly by an ABI 3730XL sequencer using BigDye Terminator (Applied Biosystems). The sequence data were assembled into contiguous sequences using the software ATGC (Genetyx, Tokyo, Japan), and sequence alignments with the consensus sequence of accession number AB513134 were generated by a progressive pairwise global alignment method using the same software.

It remains to be elucidated how Tax and HBZ contribute to the viral tumorigenesis or how the integration site of the virus affects its gene expression property. We are conducting molecular biological analysis on this cell line to elucidate its mechanistic properties for hematologic immortalization ability.

### Accession number(s).

The genome sequences of these HTLV-1 proviral genomes have been deposited in GenBank under accession numbers LC183873 (for OATL9A, located in chromosome 1) and LC378575 (for OATL9B, located in chromosome 15).
